# The peripheral epigenome predicts white matter volume contingent on developmental stage: An ECHO study

**DOI:** 10.21203/rs.3.rs-4139933/v1

**Published:** 2024-04-19

**Authors:** Sophie. Spencer, Samantha A. Harker, Fatoumata Barry, Jennifer Beauchemin, B. Blair Braden, Phoebe Burton, Viren D’sa, Daphne Koinis-Mitchell, Sarah E. Mennenga, Sean C.L. Deoni, Candace R. Lewis

**Affiliations:** Arizona State University; Arizona State University; Rhode Island Hospital, Brown University; Rhode Island Hospital, Brown University; Arizona State University; Rhode Island Hospital, Brown University; Rhode Island Hospital, Brown University; Rhode Island Hospital, Brown University; Arizona State University; Rhode Island Hospital, Brown University; Arizona State University

## Abstract

Epigenetic processes, including DNA methylation, are emerging as key areas of interest for their potential roles as biomarkers and contributors to the risk of neurodevelopmental, psychiatric, and other brain-based disorders. Despite this growing focus, there remains a notable gap in our understanding of how DNA methylation correlates with individual variations in brain function and structure. Additionally, the dynamics of these relationships during developmental periods, which are critical windows during which many disorders first appear, are still largely unexplored. The current study extends the field by examining if peripheral DNA methylation of myelination-related genes predicts white matter volume in a healthy pediatric population [N = 250; females = 113; age range 2 months-14 years; *M*_age_ = 5.14, *SD*_age_ = 3.60]. We assessed if DNA methylation of 17 myelin-related genes predict white matter volume and if age moderates these relationships. Results highlight low variability in myelin-related epigenetic variance at birth, which rapidly increases non-linearly with age, and that DNA methylation, measured at both the level of a CpG site or gene, is highly predictive of white matter volume, in early childhood but not late childhood. These novel findings propel the field forward by establishing that DNA methylation of myelin-related genes from a peripheral tissue is a predictive marker of white matter volume in children and is influenced by developmental stage. The research underscores the significance of peripheral epigenetic patterns as a proxy for investigating the effects of environmental factors, behaviors, and disorders associated with white matter.

## Introduction

The emerging field of neuroimaging epigenetics aims to identify how epigenetic profiles measured from peripheral samples relate to brain structure and function. Epigenetics is a hypernym used to describe various biological mechanisms that regulate gene transcription, such as histone modifications, chromatin modeling, regulatory RNAs, and DNA methylation (DNAm), by modulating gene expression outside of the nucleotide sequence^1^. Neuroimaging is an *in vivo* technique capable of relaying structural and functional brain mapping^2^. Thus, the cross-disciplinary application of epigenetics and neuroimaging can expand understanding of how the peripheral epigenome relates to brain structure and function.

The vast majority of current human studies that examine links between behavior and DNAm are conducted with peripheral samples, such as blood, saliva, or buccal cells. Within this design, an assumption is made that the peripheral epigenome is being used as surrogate tissue for the brain and that it is in some way reflecting brain DNAm. Many studies have reported strong correlations between post-mortem brain DNAm and peripheral DNAm within the same participants^3–7^. Three hypotheses have been proposed regarding the association between peripheral DNAm and behaviors or brain-related phenotypes: 1) the observed peripheral DNAm does not reflect the brain; 2) early environmental impacts on DNAm may occur in common precursor cells to the peripheral cells and brain, causing similar DNAm profiles across tissue types; and 3) circulating molecules such as hormones, insulin, and miRNAs, in response to an external factor, will cause the same DNAm changes in peripheral cells and brain, thus making peripheral tissue a reflection of brain tissue^8^. However, these three models are not mutually exclusive, and it is likely that the relationships between peripheral- and brain-epigenetic profiles represent a combination of the three postulated hypotheses. Interestingly, the most recent study noted that peripheral reflection of brain DNAm appears to be region specific, and that defining these relationships will accelerate neuroepigenetic research^9^. Thus, investigating peripheral-brain relationships in well-defined systems is crucial to providing biological relevance of the peripheral epigenome, which will then determine if and where the peripheral epigenome may be measured and biomarker for brain structure and function or brain based disorders.

A strength of neuroimaging epigenetics studies is the ability to associate peripheral epigenetic patterns with temporally linked brain information^10^, thus allowing for complementary mapping of epigenetic associations with the brain to the information gleaned from post-mortem epigenetic brain studies. Further, neuroimaging epigenetic methods provide the opportunity for longitudinal and prospective studies with large sample sizes that are not possible with post-mortem brain tissue. These types of cohorts and datasets will be more informative in elucidating the biological pathways linking early experiences, such as early life trauma, with later behavior by providing two theoretically linked endophenotypes: epigenetics and the brain.

The existing few studies in the field of neuroimaging epigenetics have widely focused on adult and clinical populations. A 2023 review published in *Biological Psychiatry* reported that only 21% of neuroimaging epigenetic studies explored developmental cohorts, and only 5% in childhood^11^. Even fewer neuroimaging epigenetic studies in developmental cohorts consider typical development; most examine environmental associations or clinical samples^11^. Additionally, only ten longitudinal cohorts that track both DNAm and MRI in pediatric populations were noted in a systematic review^10^. Because DNAm is dynamic throughout life, especially in the early years, associations with the brain may differ across developmental stages^12,13^. As such, studying DNAm associations with MRI metrics during development provides a unique opportunity to assess dynamic peripheral epigenetic correlates that may change over time or provide valuable biological insights predictive of brain disorders that develop later.

MRI assessment of white matter volume (WMV) can provide information on myelination, a critical component of healthy brain development and healthy brain maintenance throughout life. White matter is a lipid-rich subcortical tissue that comprises roughly half of the total human brain volume and is essential for effective neuronal communication. Oligodendrocytes are glial cells responsible for the synthesis of the myelin sheath, which supports and insulates neuronal axons. WMV increases markedly in early childhood, then levels off but remains plastic throughout life^14^. Disruptions in white matter structures have been consistently implicated in the pathophysiology of various neurological and psychological disorders^15,16^. Importantly, white matter development in the first five years of life is highly correlated with cognitive maturation and abilities imperative for life-long function^17^. Additionally, its degradation, as measured by MRI hyperintensities, is associated with stressful life events, early life socioeconomic status, sexual assault, psychiatric and neurodegenerative diseases, and cognitive decline in aging^18–23^.

Actions of myelinating-gene products guide white matter microstructure, mechanistic formation, and white matter diseases of the brain^17,19^. By definition, genetic variants and/or differing levels of expression in myelinating genes may greatly impact the development and maintenance of white matter. Despite the important role of gene products in myelination development and maintenance, no studies have explored peripheral DNA methylation of white matter genes in association with WMV during development.

The current study extends the field of neuroimaging epigenetics by examining: 1) the relationship between DNAm of myelin-related genes and age; 2) if peripheral DNAm of myelin-related genes can predict brain WMV in a healthy pediatric population; and 3) if the relationship between DNAm of myelin-related genes and WMV differs across development. Specifically, we assess DNAm variance and DNAm levels across 17 genes that are expressed in oligodendrocytes and one neuronal gene (*NRG1*) that have been previously associated with white matter structure in adult schizophrenia patients^24^. We hypothesize that DNAm of myelination-related genes measured from buccal cells will predict WMV in a healthy pediatric population.

## Methods

### Parent Study

This study was based on a subset of participants prospectively followed as part of the Environmental Influences on Child Health Outcomes (ECHO) Program. ECHO is a consortium of 69 established pediatric cohort studies collecting new data under a common protocol since 2019^25^, with the primary aim of studying the effects of early-life environmental exposures on child health. The current study included one ECHO cohort in the analyses. Single and cohort-specific institutional review boards monitored human subject activities and the centralized ECHO Data Analysis Center. All participants provided written, informed consent.

Eligibility criteria for the parent study included: mothers >18 years old, term gestation of 37-41 weeks, healthy singleton pregnancy, no evidence of uncontrolled medical conditions (i.e., hypertension, pre-eclampsia, uncontrolled diabetes) or medical conditions that could potentially impact the safety of a participant during a study visit, no history of major psychiatric illness, English speaking, consent to baby brain imaging, and the longitudinal nature of the study; infants had no significant congenital anomalies; and infants had no history of neurological trauma or disorder (e.g., epilepsy). The inclusion criteria for the subset used in this study included participants who provided a saliva sample and an MRI scan within 365 days (*M*_days_apart_ = 28.48; *SD*_days_apart_ = 69.5).

### Demographics

Demographic information was collected by parent reports. The study sample included youth [N = 250; females = 113; age range 2 months-14 years; *M*_age_ = 5.14, *SD*_age_ = 3.60]. Sample demographics are summarized in Table 1, and a flowchart on how the analytic sample was derived can be found in supplemental materials.

### Saliva Collection and DNA Isolation

Saliva was collected from participants in the lab or at home using Oragene (DNA Genotek, Ottawa, Ontario, Canada) saliva collection kits. DNA was extracted with a standard isolation kit (DNA Genotek’s PT-L2P-5). Sample yield and purity were assessed spectrophotometrically using NanoDrop ND-1000 methods (ThermoScientific, Wilmington, DE).

### DNA Methylation

The DNA was bisulfite treated with the EZ-96 DNA Methylation Kit (Zymo Research) according to Illumina’s recommended deamination protocol. Genomic annotations and probes for the EPIC array were acquired through the support website of Illumina Inc. (https://support.illumina.com/). The Illumina Infinium MethylationEPIC array BeadChip (850K) was carried out at either the home institution (n = 151) or by the Epigenomic Services from Diagenode (n = 271; Cat nr. G02090000). Due to power constraints, we chose a hypothesis-driven method instead of a genome-wide analysis. We investigated 17 genes that are expressed in oligodendrocytes and one neuronal gene (*NRG1*) that have been previously associated with white matter structure^24^. All CpG sites annotated to genes of interest per the EPIC platform were used in analyses. Genes of interest were chosen based on their association with white matter structure in previous literature^24^. Location designation was used as a covariate in all analyses. The probe sequences were aligned to the human reference genome (hg19) with two types of probes: Infinium I (2 probes/locus) or Infinium II (1 probe/locus). Illumina describes that the location of the CpG is relative to the CpG island with the sequence of annotation: shores being 0-2 kb from islands, shelfs being 2-4 kb from islands, and open sea regions isolated CpG sites in the genome that do not have a specific designation. North refers to upstream (5’) of the CpG island, and south refers to downstream (3’) of the CpG island.

### DNA Methylation Data Preprocessing

Raw Intensity Data (IDAT) files were exported for preprocessing in R with the minfi package. Probes on sex chromosomes were removed with a filter, and standard quality control analyses were performed, including quantile normalization, identifying sex mismatches, and excluding low-intensity samples (*p* detection < 0.01)^26^. Three samples did not pass our quality control pipeline due to their low intensity. Using the Minfi package, the data were normalized and annotated with Illumina CpG site probe names. Using the R package EpiDISH (Epigenetic Dissection of Intra-Sample Heterogeneity, 3.8) Robust Partial Correlation (RPC) method, we estimated the proportion of epithelial cells per sample. Cell count was included as a covariate in all analyses. M-values were used for methylation analysis as recommended by previous research^27^.

### MRI

Imaging was performed at the Lifespan facility at Brown University. All data was collected without sedation. For infants, toddlers, and young children (< 4-5 years of age), imaging was performed immediately following feeding, during normal naptime, or at bedtime. Participants were also mildly sleep-deprived on the scan day (woken early or missed a nap). Older children who were able to remain motionless throughout the scan were scanned during the day or evening. While in the scanner, the infant or child was continuously monitored with an integrated Siemens pulse oximetry system. They were also visually monitored by a research team member at the scanner suite and by the scanner operator via an infrared camera. At the first sight of the child waking or at their parent’s discretion, they were immediately removed from the scanner. Structural T1 images were processed using FreeSurfer^28^. Total white matter measurements were divided by 10,000 to adjust the scale of the variable and enable easier interpretation.

### Statistics

#### WMV and DNAm relationships with age

As a data quality control measure, we evaluated known non-linear associations between WMV and age in early development. Further, we compared linear and quadratic regression models on the relationship between DNAm of myelin-related genes and age. Previous findings on WMV across age^14^ and DNAm across age^29^ guided our analysis of these variables. R^2^ values and the percent increase in R^2^ between linear and quadratic models were compared. A > 5% increase in R^2^ values between linear and quadratic results constituted a better prediction model^29^.

#### Principal component analysis (PCA)

PCA is a commonly used method to detect patterns in DNAm data^30,31^. PCA is a dimension reduction technique used to develop a smaller number of variables, called principal components (PCs). The first PC (PC1) accounts for most of the variance in the observed variables of a data set^32^. For all genes of interest, annotated CpG sites were transformed into PCs. Next, CpG sites with an absolute loading value less than 0.3 on the PC1 were removed from the analysis and PCs regenerated. The PC1 was used in later analyses. PCA was conducted in IBM^®^ SPSS^®^ (Statistical Package for the Social Sciences). Our group has used this method for DNA methylation analyses in prior studies^33–35^.

#### Principal components regression model fit

Principal component regression (PCR) was used on each individual gene to determine the optimal number of PCA components for model fit in predicting WMV. PCR is a combination of principal components analysis and multiple linear regression. PCR creates principal components from explanatory variables, then, with a user-determined number of PCs, attempts to predict the outcome variable in the subsequent regression analysis. Data from a random sample of 125 participants was used as a training dataset. Data from the remaining 125 participants was used as a test dataset. We compared model fit using 1, 2, 5, 10, and the maximum number of PCs predicting WMV for each gene of interest. Root mean square error (RMSE) was used to determine model fit and assess the optimal number of PCs to use in the next analyses.

#### Multiple linear regression

To account for the strong effect of age on DNAm and WMV through development, we employed two statistical approaches to ensure robust results. First, we used group-mean centering to remove the variance associated with age in the raw epigenetic and WMV values (early childhood < 6 years old and late childhood > 6 years old). Secondly, chronological age was used as a covariate in all analyses. PCR determined the best model fit was achieved by including only the first component for each gene (PC1). Multivariate linear regression models were run separately for each gene with WMV as the outcome and PC1, age, PC1xAge, sex, estimated cell count, array batch, and days between saliva collection and brain scan as predictor variables. We also computed the average PC1 value across all genes as a predictor value. Additionally, the covariates were used in multivariate linear regression models for each CpG site on all genes of interest with 0.05 FDR correction per gene. Significant interaction effects were explored with correlation analyses to understand the strength and direction of the relationship between WMV and DNA methylation within early childhood (< 6 years old) and later childhood (> 6 years old).

#### Bootstrap resampling

Bootstrap resampling was employed to estimate a sampling distribution of the correlation between WMV and DNA methylation PC1 for each gene of interest using the “boot” package in R. For each iteration of the bootstrapping process, a random sample of the same size as the original dataset was drawn with a replacement from the dataset. This process was repeated 10,000 times to obtain stable estimates of the sampling distribution. Confidence intervals were constructed based on the percentile method to estimate the range of plausible values for the population parameter.

#### Saliva DNA methylation correlated with brain DNA methylation

DNAm levels of CpG sites annotated to myelin-related genes were compared to the same CpG sites measured in brain tissue. Brain DNAm values were obtained from the Allen Brain Atlas BrainSpan data, a publicly available dataset based on the Infinium HumanMethylation450 BeadChip. The BrainSpan dataset provided DNAm values (N = 16; *M*_age_ = 8.3, *SD*_age_ = 10.0) from 16 varying brain regions per participant (N_samples_ = 91). To conduct correlation analyses, we calculated an average methylation value across participants for all CpG sites annotated to our genes of interest. Methylation values for brain data were averaged across participants and across brain regions. An average of all CpG sites located on both the 450k (brain) and EPIC (saliva) datasets were correlated.

## Results

### WMV and DNAm Relationships with Age

**Figure 1:**
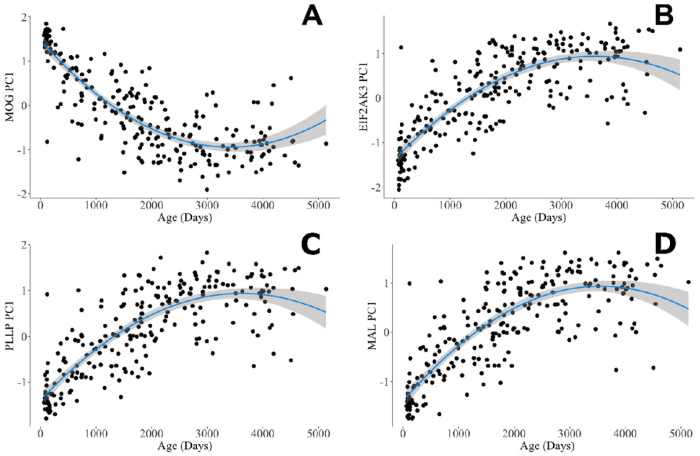
DNA methylation of myelin-related genes across development The first principal component of *MOG*, *EIF2AK3*, *PLLP*, and *MAL* DNA methylation changes quadratically across age in a cross-sectional healthy pediatric cohort. Gray shading represents the standard error of the coefficient estimate in the regression model. These genes were chosen for depiction as they had the largest r^2^ values *MOG* (Age β = −0.14, Age^2^ β = 0.002; *p’s* < 0.00001), *EIF2AK3*(Age β = 0.13, Age^2^ β = −0.001; *p’s* < 0.00001), *PLLP* (Age β = 0.13, Age^2^ β = −0.002; *p’s* < 0.00001), *MAL* (Age β = 0.13, Age^2^ β = −0.0018498; *p’s* < 0.00001). DNA methylation variance between-individuals is generally low in the first year of life and becomes more variable as age increases.

We reproduce known non-linear associations between age and WMV in development (Supplemental Figure 1). Further, we demonstrate a non-linear relationship between age and DNAm variance across myelin-related genes (Figure 1). R^2^ values and the percent increase in R^2^ between linear and quadratic models are reported in Table 2. For DNAm and age, quadratic models produced an average 21.2% increase in R^2^ values compared to linear models.

### Principal Components Analysis

The number and location of retained CpG sites in the PCA for all 17 genes of interest are detailed in Table 3. Results demonstrate that certain CpG sites from differing intragenic locations (e.g., islands, shores, and shelves) fluctuate together. Additionally, CpG sites in open sea locations, whose functional consequences are not well understood, also fluctuate with intragenic CpG sites.

### Model fit Using Principal Components Regression

Generally, models using 10 PCs outperformed models using fewer PCs (Table 4). However, the gain in model fit between using only the PC1 and using ten PCs appears inconsequential (generally the gained fit was 0.001), suggesting that studies with small- to medium-sized sample sizes can reliably use the PC1 to avoid model overfitting.

### DNAm PC1 Predicting WMV

**Figure 2:**
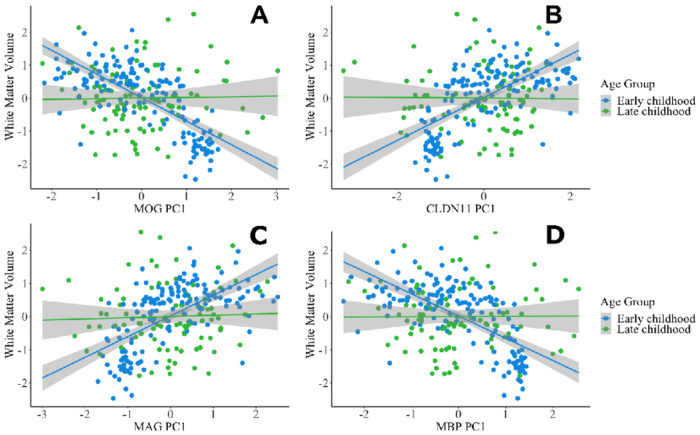
DNA methylation of myelin-related genes predicts white matter volume in early but not late childhood Regression models included age as an interaction term. PC1-age interactions significantly predicted total white matter volume. *MOG*(PC1 β = −1.39; PC1xAge β = 0.10), *CLDN11* (PC1 β = 1.44; PC1xAge β = −0.10), *MAG* (PC1 β = 1.39; PC1xAge β = −0.10), *MBP* (PC1 β = −1.47; PC1xAge β = 0.10) had the largest interaction effect sizes (all p’s < 0.00001) on total white matter volume. Gray shading represents the standard error of the coefficient estimate in the regression model.

Results from multiple linear regressions are summarized in Table 5. Overall, the main effect of the DNAm PC1 of every gene significantly predicted WMV. Additionally, every PC1-age interaction was significant, suggesting the relationship between PC1 and WMV is moderated by age (Figure 2). Lastly, an average of all PC1s significantly predicted WMV (*p* = 8.52e-11), with a significant interaction with age (*p* = 0.000272). Following significant interaction terms, post-hoc subgroup correlation analyses revealed that all genes are significantly correlated with WMV (all *p*’s < 0.001) in early childhood (< 6 years old), whereas only *MOBP* was significantly correlated with WMV (r = 0.225, p = 0.02) in late childhood (> 6 years old).

### Bootstrap Resampling

The 95% confidence interval of the estimated distribution of correlation values between WMV and DNA methylation obtained through 10,000 resampling iterations contained the sample correlation value for all genes of interest (Table 6). These results support the reliability of our findings.

### DNAm Levels at CpG Sites Predicting White Matter

Table 7 summarizes the results of multivariate linear models with DNAm from single CpG sites predicting WMV. Generally, CpG sites located in open sea regions tended to be good predictors of WMV while CpG sites located on shelves did not. Seven of the 17 genes assessed did not have a single CpG site located on an island that predicted WMV.

### Correlation between Buccal and Brain DNA Methylation

**Figure 3:**
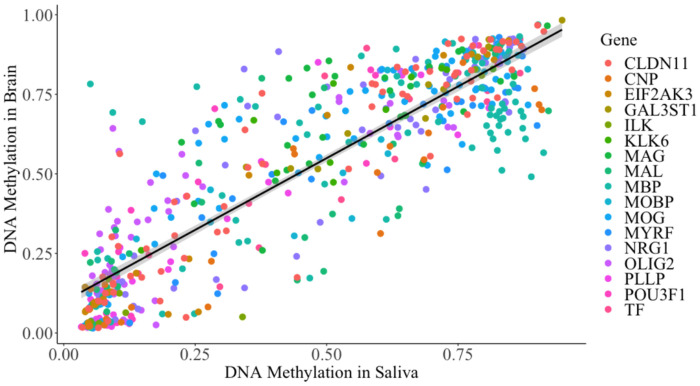
Correlation between salivary DNA methylation and brain DNA methylation Each data point represents average DNA methylation at CpG sites across either brain or buccal samples and colored by their annotated gene. Gray shading represents the standard error of the coefficient estimate in the regression model. Brain DNA methylation data was extracted from the Allen Brain Atlas Brainspan Average DNA methylation in buccal and brain are significantly correlated (*r* = 0.882, *p* < 0.0001).

Table 8 summarizes the correlation between the CpG DNAm measured in this study and publicly available brain DNAm for all 17 genes of interest. Overall, most CpG sites had large correlations between saliva and brain-derived DNAm. Assessed together, all CpG sites had a large correlation between saliva and brain (*r* = 0.88, *p* = 0.0001; Table 8). Figure 3 displays the correlation between DNA methylation measured in brain tissue and this study in buccal cells for all overlapping CpG sites between the studies.

## Discussion

Results from this study provide compelling evidence that DNAm of genes associated with myelination, when measured in buccal cells, may serve as predictive markers for WMV in a normative pediatric cohort. Importantly, results demonstrate that the relationship between WMV and peripheral epigenetics is contingent upon the developmental phase, such that this relationship appears to subside after early childhood, underscoring the intricate interplay between epigenetic modification and neurodevelopment. Furthermore, our approach provides evidence that the variance in DNAm across the gene body (as gauged by PCA), alongside individual CpG methylation levels throughout the gene and in flanking non-coding regions, are both instrumental metrics of DNA methylation given their capacity to prognosticate anatomical phenotypes downstream. Such findings should inform future research to further probe our understanding of the biological functionality of DNA methylation beyond island regions.

Myelin-related genes play diverse and important roles in white matter development and maintenance. For example, the *MOG* gene plays a crucial role in the structural component of the myelin sheath through its creation of the myelin oligodendrocyte glycoprotein^36–38^. Genetic variations of *MOG* have been associated with decreased total WMV in children with obsessive-compulsive disorder^38^. Oligodendrocyte cell body size has been related to increased expression of *OLIG1* and *MOG* mRNA levels in post-mortem brain samples from adults with Major Depressive Disorder^37^. Notably, *MYRF* expression has been found to proliferate during the premyelinating and myelinating stages of oligodendrocyte development^39^. Concordantly, *MYRF* is known to activate the expression of genes related to myelin structure, such as *MOG, MBP, MAG*, and *PLP*^40^. *MYRF*, therefore, is not only crucial in promoting the maturation of oligodendrocytes, but it is also important for the maintenance of oligodendrocytes even after development and maturation are complete. Koenning and colleagues demonstrated this facet of expression through the ablation of *MYRFin* knockout mice, causing degeneration of myelin sheaths in the adult CNS and decreased expression of *PLP, MAG, MBP*, and *MOG*^41^. Despite *MYRF* importance across the lifespan, our data suggests that peripheral DNA methylation of this gene, among others, is more reflective of WMV in young, but not older, children. Future studies are needed to characterize how the relationship between peripheral DNA methylation of myelin-related genes and WMV may change throughout the life course.

Results from this study highlight the importance of DNA methylation patterns across the gene body, as opposed to levels of DNA methylation at specific CpG sites. Using PCA analyses, we show that CpG site methylation levels from all gene regions (i.e., island, shore, shelf, and open sea) correlate, as can be seen by their loading together on the first principal component (Table 3), indicating that both intragenic CpG methylation levels and variance can predict WMV. Importantly, our group has previously used this method and found associations between parenting styles and epigenetics of hypothalamic-pituitary-adrenal axis genes^35^ and immune genes^34^ in a pediatric cohort. Not only did we find that social exposures predicted PC1s, we further demonstrated that PC1 predicts diurnal cortisol slope^35^ and physical health^34^, highlighting the exposure to epigenome to physiology pathway. Importantly, we further confirmed the impact of environment by controlling for genetics in a monozygotic twin difference design, in which the co-twin difference in the first principal components of dopaminergic genes predicted the co-twin difference in cognitive function^33^. Taken together, gene-level DNA methylation summary scores that capture variance across the gene body appear to be an effective strategy for assessing relationships with social exposures, behavior, and physiology.

Most studies of DNAm in relation to brain and behavior have assumed that functionally important DNAm will occur in promoters and that most DNAm underlying brain and behavioral health occur in CpG islands. However, prior research suggests that CpG methylation in shores is also anti-correlated with gene expression levels^43,44^ and that the methylation status of CpGs in the gene body can correlate with gene expression in the absence of DNAm methylation changes in promoter regions^45^. A meta-analysis of genome-wide epigenomic and gene expression data from cell lines describes a bell-shape distribution, i.e., the lowest levels of intragenic DNA methylation correspond to both the lowest and highest expressed genes, while the highest methylation levels are associated with genes expressed at intermediate levels^46^. Additionally, intragenic methylation levels may also affect downstream biology by regulating alternative transcripts, alternative splicing, non-coding RNAs, and transposable elements^47^. The importance of intragenic DNAm is consistent with cancer studies^43,44,48^ and a more recent study implicating DNAm at CpG shores in the onset of Alzheimer’s disease^49^. Thus, we call for more studies to incorporate the assessment of intragenic CpG sites within studies of early life stress exposure, brain, and behavior.

Prior research has documented consistent trajectories of both DNAm and myelination development in early life. Specifically, both DNAm and myelination development are more rapid in early life compared to later in life^50,51^. Both myelination and DNAm follow a characteristic pattern of change^50,51^ and are thought to be especially sensitive to environmental impacts during early life^51,52^. As such, both systems mature alongside the development of various complex behavioral outcomes^17,53^, and alterations in both are consistently found across neurodevelopmental, psychiatric, and neurodegenerative disorders. Here, we identified a developmental window in which peripheral epigenetic signatures are able to predict WMV. Specifically, these results suggest that myelin-related gene DNAm measured in buccal cells may serve as predictors for WMV in infancy and early childhood but not in middle or late childhood. These results are interesting as the developmental window maps well onto the rapid development and plasticity of both myelination and DNAm. However, we are careful to note the possibility that later events, such as adolescence or trauma, may introduce another opportunity for peripheral signatures to predict brain structure. Future research with longitudinal cohorts consisting of a tight age range could further evaluate these questions.

Generally, at age-variable CpG sites, DNAm levels tend to decrease with age while also increasing in between-person variability^50^. However, much of the prior research has assessed DNAm change over time using linear models, but a recent study demonstrated that quadratic models may better fit the change over time in early development^29^. Here, assessing myelination-related gene DNAm, our results are consistent with rapid non-linear change and increased between-person variability after the first year of life^29,50,54^. Further, because we used principal components, we show that variance across a gene, not just levels, of DNAm increases with age. Such results suggest that quadratic and linear statistical models should be compared for the best model fit in epigenetic change across age, particularity in early development.

There are limitations to this study that are important to note. Notably, the inclusion of another peripheral sample would have improved this study, as it is plausible that another tissue type may more accurately predict brain structure metrics. Importantly, array-based methods of measuring DNAm cannot differentiate between methylation and hydroxymethylation, which play differing roles in transcription regulation. Because our study did not measure downstream implications of DNAm, such as RNA or protein levels, we cannot ascertain the functional impact of DNAm on gene transcription. Additionally, our study is limited by a cross-sectional design and would benefit from reproduction in a longitudinal cohort. However, our cross-sectional cohort is more diverse than typical neuroimaging^55^ and genomic studies^56^, consisting of 32% non-White and 20% Hispanic participants. While beyond the scope of this study, future studies should assess if racial, ethnic, or other social detriments to health, such as socioeconomic status, moderate the relationships between WMV and peripheral epigenetics. Lastly, because this is a self-selected healthy population from a single geographical location, it is unknown if the results will generalize to other populations or ages.

Synoptically, these results highlight a strong relationship between the peripheral DNAm of myelin-related genes and WMV in young children but not older children. We demonstrate low variability in myelin-related epigenetic variance at birth, which rapidly increases non-linearly with age when measured in a peripheral sample. Potentially, increased variance with age is at least partially reflecting individual differences in social determinants of health known to impact the epigenome in development, such as socioeconomic status, nutrition, toxin exposures, and stress. Importantly, we also demonstrate that peripheral epigenetic profiles of myelin-related genes are highly correlated with profiles measured in the brain from an independent cohort. Taken together, these results suggest that peripheral myelinating-epigenetic profiles may serve as indicators of developmental milestones or developmental delays due to either genetic or environmental impacts on WMV development^29,54^. These results corroborate previous evidence that the peripheral epigenome can predict brain structure/function and behavior. The question of whether peripheral tissues can accurately reflect the epigenetics of brain tissue is an ongoing research question and needs to be continuously examined. Our study, however, suggests that the well-known “tissue issue” may be less of an issue after all within certain systems, and perhaps a more complex and further refined composite of peripheral DNAm may one day serve as a biomarker of overall brain health.

## Figures and Tables

**Table 1.** Study sample descriptive statistics

Age		
Mean ± SD	5.14 ± 3.60	
Range	2m - 14y	
Sex (%)		
Male	55	
Female	45	
Days in between brain scan and saliva collection		
Mean ± SD	28.48 ± 70.3	
Self-Reported Race (%)	%	n
American Indian or Alaska Native	0.4	1
Asian	2.8	7
Black or African American	7.6	19
Mixed Race	12.8	32
Native Hawaiian or Pacific Islander	0.4	1
Other, Unknown, Declined, or Missing	8.4	21
White	67.6	169
Self-Reported Ethnicity (%)	%	n
Non-Hispanic/Latino	73.2	183
Hispanic/Latino	20.4	51
Declined or Missing	6.4	16

**Table 2.** Model comparison of DNA methylation in development

Gene	R^2^		% change
	Linear*	Quadratic*	
***MOG***	0.57	0.71	23.59%
***MBP***	0.56	0.68	21.44%
***EIF2AK3***	0.58	0.68	18.07%
***MYRF***	0.50	0.59	18.89%
***NRG1***	0.52	0.63	20.70%
***GAL3ST1***	0.47	0.52	9.48%
***PLLP***	0.57	0.67	17.79%
***MAL***	0.57	0.68	19.27%
***OLIG2***	0.54	0.63	15.55%
***ILK***	0.20	0.27	35.96%
***MAG***	0.55	0.64	17.03%
***POU3F1***	0.35	0.43	21.22%
***MOBP***	0.19	0.24	31.15%
***CNP***	0.50	0.58	15.92%
***TF***	0.47	0.55	17.45%
***KLK6***	0.52	0.63	20.80%
***CLDN11***	0.55	0.65	18.85%

* all models p < 0.0001

**Table 3.** Number and location of retained CpG sites in principal components analyses

Gene	Island	North Shelf	North Shore	Open Sea	South Shelf	South Shore	Total
*MOG*	0	1	6	35	3	2	47
*MBP*	14	10	6	51	5	12	98
*EIF2AK3*	3	1	5	11	1	2	23
*MYRF*	4	5	2	10	6	2	29
*NRG1*	3	0	1	141	0	2	147
*GAL3ST1*	2	0	2	18	0	1	23
*PLLP*	3	2	2	25	1	2	35
*MAL*	6	2	3	23	2	5	41
*OLIG2*	18	2	6	17	1	6	50
*ILK*	1	0	2	0	1	8	12
*MAG*	10	7	4	17	5	3	46
*POU3F1*	15	0	2	11	3	2	33
*MOBP*	3	0	7	18	1	3	32
*CNP*	5	0	2	5	1	1	14
*TF*	3	1	8	11	1	5	29
*KLK6*	0	0	0	21	0	0	21
*CLDN11*	11	2	2	70	4	5	94

**Table 4:** Test of model fit for number of principal components to predict white matter volume

Gene*	1 component		2 components		5 components		10 components		Max components	
	RMSE	Standardized RMSE	RMSE	Standardized RMSE	RMSE	Standardized RMSE	RMSE	Standardized RMSE	RMSE	Standardized RMSE
***MOG*** (79)	0.917	0.020	0.916	0.020	0.865	0.019	0.822	0.018	1.151	0.026
***MBP***^ (111)	0.929	0.021	0.936	0.021	0.880	0.020	0.852	0.019	1.430	0.032
***EIF2AK3*** (56)	0.913	0.020	0.916	0.020	0.876	0.019	0.852	0.019	1.231	0.027
***MYRF*** (60)	0.937	0.021	0.952	0.021	0.906	0.020	0.892	0.020	1.101	0.024
***NRG1***^ (111)	0.935	0.021	0.938	0.021	0.845	0.019	0.831	0.018	1.197	0.027
***GAL3ST1*** (36)	0.962	0.021	0.945	0.021	0.940	0.021	0.900	0.020	1.054	0.023
***PLLP*** (58)	0.931	0.021	0.933	0.021	0.863	0.019	0.882	0.020	1.011	0.022
***MAL*** (70)	0.927	0.021	0.930	0.021	0.850	0.019	0.802	0.018	1.289	0.029
***OLIG2*** (84)	0.942	0.021	0.947	0.021	0.895	0.020	0.911	0.020	1.640	0.036
***ILK*** (26)	0.963	0.021	0.908	0.020	0.918	0.020	0.893	0.020	0.980	0.022
***MAG*** (57)	0.939	0.021	0.943	0.021	0.878	0.020	0.891	0.020	1.290	0.029
***POU3F1*** (43)	0.942	0.021	0.924	0.021	0.866	0.019	0.874	0.019	0.934	0.021
***MOBP*** (49)	0.978	0.022	0.961	0.021	0.866	0.019	0.852	0.019	0.984	0.022
***CNP*** (28)	0.929	0.021	0.931	0.021	0.905	0.020	0.918	0.020	0.903	0.020
***TF*** (42)	0.952	0.021	0.953	0.021	0.932	0.021	0.957	0.021	1.147	0.025
***KLK6*** (29)	0.945	0.021	0.930	0.021	0.905	0.020	0.939	0.021	0.980	0.022
***CLDN11***^ (111)	0.937	0.021	0.949	0.021	0.840	0.019	0.842	0.019	1.754	0.039

* Parentheses next to gene name indicate the maximum components.

^ Sample size restricted them model to a maximum of 111 components to be analyzed. Actual maximum components were MBP = 169, NRG1 = 200, CLDN11 = 149

**Table 5.** DNA methylation predicting white matter volume moderated by age

Gene	Main Effect				Interaction	
	PC1		Age			
	β	*p*	β	*p*	β	*p*
***MOG***	−1.39	**< 2e-16**		**4.360E-06**	0.10	**3.160E-14**
***MBP***	−1.47	**< 2e-16**	0.15	**2.030E-07**	0.10	**3.700E-13**
**EIF2AK3**	1.32	**< 2e-16**	0.14	**2.810E-07**	−0.09	**5.060E-11**
***MYRF***	1.26	**< 2e-16**	0.14	**4.790E-07**	−0.07	**3.350E-07**
***NRG1***	−1.48	**< 2e-16**	0.14	**1.490E-06**	0.11	**5.380E-13**
***GAL3ST1***	0.84	**1.120E-09**	0.07	**1.060E-02**	−0.06	**4.790E-05**
***PLLP***	1.37	**< 2e-16**	0.15	**1.070E-06**	−0.10	**8.620E-12**
***MAL***	1.36	**< 2e-16**	0.14	**6.210E-07**	−0.09	**4.640E-12**
***OLIG2***	1.22	**4.040E-15**	0.14	**6.270E-06**	−0.08	**3.120E-08**
***ILK***	−0.84	**1.390E-09**	0.05	**4.390E-02**	0.06	**2.510E-04**
***MAG***	1.39	**< 2e-16**	0.15	**4.790E-07**	−0.10	**3.670E-11**
***POU3F1***	1.14	**2.010E-15**	0.12	**7.410E-06**	−0.05	**3.590E-04**
***MOBP***	−0.55	**1.390E-05**	0.01	**5.435E-01**	0.08	**2.050E-06**
***CNP***	1.07	**8.160E-14**	0.13	**1.290E-05**	−0.07	**2.850E-06**
***TF***	−1.21	**9.110E-15**	0.10	**7.290E-04**	0.11	**1.080E-11**
**KLK6**	−1.26	**4.450E-16**	0.11	**5.430E-04**	0.11	**2.260E-12**
***CLDN11***	1.44	**< 2e-16**	0.15	**5.990E-07**	−0.10	**2.550E-13**

**Table 6.** Estimated distribution of correlation values between WMV and DNA methylation

Gene (PC1)	Sample correlation	95% Interval	Sample correlation contained in estimated distribution?
***MOG***	−0.73	−0.79 : −0.67	Yes
***MBP***	−0.70	−0.76 : −0.63	Yes
***EIF2AK3***	0.70	0.62 : 0.76	Yes
***MYRF***	0.64	0.56 : 0.71	Yes
***NRG1***	−0.67	−0.73 : −0.60	Yes
***GAL3ST1***	0.56	0.47 : 0.64	Yes
***PLLP***	0.68	0.61 : 0.74	Yes
***MAL***	0.69	0.62 : 0.76	Yes
***OLIG2***	0.68	0.61 : 0.74	Yes
***ILK***	−0.48	−0.56 : −0.39	Yes
***MAG***	0.66	0.59 : 0.72	Yes
***POU3F1***	0.54	0.45 : 0.62	Yes
***MOBP***	−0.43	−0.53 : −0.33	Yes
***CNP***	0.63	0.55 : 0.70	Yes
***TF***	−0.64	−0.70 : −0.56	Yes
***KLK6***	−0.67	−0.73 : −0.60	Yes
***CLDN11***	0.68	0.61 : 0.74	Yes

**Table 7.** Relationships between DNA methylation of CpG sites, site location, and white matter volume

Gene	Total CpGs	Passed FDR (%)	+ β	− β	Island	North Shelf	North Shore	Open Sea	South Shore	South Shelf
***MOG***	74	11 (15)	4	7	0	0	4	**9**	1	0
***MBP***	164	29 (18)	17	12	2	3	2	**7**	2	2
***EIF2AK3***	50	10 (20)	8	2	0	0	**8**	**8**	2	2
***MYRF***	55	9 (16)	7	2	2	2	2	**5**	0	5
***NRG1***	194	36 (19)	17	19	0	0	0	**19**	0	0
***GAL3ST1***	31	2 (6)	1	1	0	0	0	**6**	0	0
***PLLP***	53	15 (28)	6	9	2	0	2	**21**	2	2
***MAL***	65	17 (26)	14	3	5	0	5	**15**	2	0
***OLIG2***	79	15 (19)	7	8	4	0	5	**9**	1	0
***ILK***	21	2 (10)	1	1	0	5	0	0	5	0
***MAG***	52	11 (21)	9	2	**6**	2	4	**6**	4	0
***POU3F1***	38	9 (24)	7	2	**11**	0	3	8	3	0
***MOBP***	44	8 (18)	2	6	2	0	2	**11**	2	0
***CNP***	23	6 (26)	2	4	**9**	0	**9**	**9**	0	0
***TF***	37	6 (16)	4	2	0	0	5	5	5	0
***KLK6***	24	8 (33)	8	0	0	0	0	**33**	0	0
***CLDN11***	144	29 (20)	18	11	1	0	1	**17**	1	0

**Table 8.** Saliva and brain CpG site correlation

Gene	*r*
***MOG***	0.91
***MBP***	0.65
***EIF2AK3***	0.97
***MYRF***	0.90
***NRG1***	0.89
***GAL3ST1***	0.97
***PLLP***	0.94
***MAL***	0.81
***OLIG2***	0.83
***ILK***	0.94
***MAG***	0.63
***POU3F1***	0.90
***MOBP***	0.90
***CNP***	0.94
***TF***	0.87
***KLK6***	0.86
***CLDN11***	0.93

Whole model r = 0.882, p < 0.0001

Single gene models all p < .0001

## Data Availability

All data used for these analyses will be released through the ECHO Data Availabile to the Scientific Community (DASH) portal.
